# An eye-tracking study of reading long and short novel and lexicalized compound words

**DOI:** 10.16910/jemr.13.4.3

**Published:** 2020-08-04

**Authors:** Jukka Hyönä, Alexander Pollatsek, Minna Koski, Henri Olkoniemi

**Affiliations:** 1 University of Turku Turku, Finland; 2 University of Massachusetts Amherst, USA

**Keywords:** Eye movement, eye tracking, saccades, microsaccades, antisaccades, smooth pursuit, scanpath, convergence, attention

## Abstract

An eye-tracking experiment examined the recognition of novel and lexicalized compound words during sentence reading. The frequency of the head noun in modifier-head compound words was manipulated to tap into the degree of compositional processing. This was done separately for long (12–16 letter) and short (7-9 letters) compound words. Based on the dual-route race model [Pollatsek et al., 4] and the visual acuity principle [Bertram & Hyönä, 2], long lexicalized and novel compound words were predicted to be processed via the decomposition route and short lexicalized compound words via the holistic route. Gaze duration and selective regression-path duration demonstrated a constituent frequency effect of similar size for long lexicalized and novel compound words. For short compound words the constituent frequency effect was negligible for lexicalized words but robust for novel words. The results are consistent with the visual acuity principle that assumes long novel compound words to be recognized via the decomposition route and short lexicalized compound words via the holistic route.

## Introduction

Novel words can be created from existing ones by combining two existing words to form a new compound word. In many languages word compounding is very productive; thus, novel compound words can be easily formed. For example in Finnish, the language used in the present study, a novel compound word can be created by simply attaching two nouns to each other, *tulkinta* (= translation) and *tehtävä* (= job), to form a novel compound word *tulkintatehtävä* (= translation job). Although a reader of Finnish is unlikely to have encountered this word before, (s)he can readily understand the intended meaning of the word by combining the meanings of the two components. This feature is not unique to Finnish, as word compounding is productive in a great number of languages (for example, in English and in Germanic languages).

Writers often create novel compound words on the fly. Thus, readers of Finnish newspapers encounter daily novel compound words. For example, at the outbreak of the corona virus pandemic, a novel compound word *viruslinko* (virus blower or virus spin) was invented to refer to bars in ski resorts where the virus spread widely among the customers. Nagy and Anderson [1] have estimated that over half of the new words students learn in English textbooks have meanings that can be derived through morphemic analysis (including derivational morphology).

In the present study, we examined how competent adult readers process novel compound words embedded in written sentences, where the prior sentence context did not constrain the meaning of the target words. Thus, even though the target compounds appeared in context, our focus was on how readers derive the meaning of novel compound words when the meaning is not constrained by prior sentence context.

### Theories of compound word recognition

By definition, novel compound words do not have an entry in the reader’s mental lexicon. Thus, its meaning has to be derived from the component meanings, for which readers have a representation in their mental lexicon. Thus, novel compound words have to be processed via their components using the (de)composition route assumed to be in use when reading existing morphologically complex words. On the other hand, as lexicalized compounds are likely to be mentally represented, they can in principle be processed as single entities via the holistic route, that is, without the need for morphemic analysis. According to the visual acuity principle proposed by Bertram and Hyönä [2, 3], compound words may be processed holistically when they are short enough to fit in the foveal area of the eyes where visual acuity is at its best. This is because all letters constituting the word are simultaneously visible to the reader. However, this is not the case with long compound words. As a result, their processing is assumed to be initiated by first accessing the initial component, followed by the access to the second component, and then to the whole word representation. To sum up, it may be predicted that all novel compound words and long lexicalized compound words are processed via the (de)composition route, while only short lexicalized compound words are recognized via the holistic route.

It is unlikely that the use of the two access routes is mutually exclusive. Pollatsek, Hyönä and Bertram [4] put forth a *dual-route parallel race model* [see also Schreuder & Baayen, 5] to explain the recognition of lexicalized compound words. According to the model, the decomposition route and the holistic route operate in parallel. Yet, the decomposition route gets a head start, as access to the first component is faster than that to the whole word (at least when compound words are long). The whole word representation is nevertheless readily accessed; the study of Pollatsek et al. [4] suggests that the timing of the activation of the whole word representation is roughly parallel to the activation of the second component.

What would the dual-route race model predict about processing novel compound words? As no whole-word representations exist for novel compounds, their meaning has to be derived by accessing the component lexemes and their meaning. Thus, the model predicts that the (de)composition route plays a greater role in processing novel than lexicalized compounds, as the use of the holistic route is not available for novel compounds.

In the present study, we tapped into the operation of the decomposition route by applying the logic developed by Taft and Forster [6]. According to their logic, if the decomposition route is in operation in compound word processing, the frequency of the compound word constituent should exert a significant effect on the recognition process. On the other hand, the holistic route is implicated by the absence of a constituent frequency effect and by the presence of a whole word frequency effect. In our study, we manipulated for noun-noun compounds the frequency of the head noun, which always appears as the right (i.e., second) constituent and is modified by the left (i.e., first) constituent. The compound word head is the kernel of the word meaning (e.g., the compound word *translation job* refers to a specific type of job). As the holistic route is not available for processing novel compounds, readers can only use the composition route to derive the word meaning. Thus, the dual-route race model predicts a larger component frequency effect for novel than lexicalized compound words. If we add to this the prediction formulated above based on the visual acuity principle, we can predict the difference in the second constituent frequency effect between novel and lexicalized compounds to be particularly pronounced for short compound words. It may be recalled that the visual acuity principle assumes short lexicalized compounds to be processed primarily via the holistic route.

Before presenting our study in more detail, we first review what is known about processing lexicalized compounds words during reading, followed by a discussion of the limited literature on reading novel compound words. We particularly focus on eye-tracking studies of reading, as they are most relevant to the present study. It should be noted that there is an extensive literature on how new words are learned in a second language and how readers incidentally acquire new vocabulary items by mere exposure to them in text. In incidental learning of new vocabulary items, the use of contextual cues is a core feature of the process [see e.g., Morris & Williams, 7]. Such studies are not reviewed here, as they are not directly relevant to the process of meaning composition out of existing lexemes the reader is familiar with and knows the meaning of. As noted above, the present study focuses on online meaning composition based on the word’s morphological structure in the absence of supportive contextual cues.

### Eye-tracking studies of compound word reading

Several studies have investigated reading of lexicalized compound words in sentence context [for a review, see Hyönä, 8]. They have primarily been conducted in English and Finnish with two-constituent compounds. A key question has been to assess the extent to which the process is compositional or holistic in nature. Following the logic briefly described above [Taft & Forster, 6], the researchers have manipulated the frequency of the first or second constituent or that of the whole word. In what follows, we review the results separately for long and shorter compound words.

As reviewed by Hyönä [8], most studies with long compounds (≥ 11 letters) have observed a reliable effect (an effect size of 47–87 ms) of first-constituent frequency in gaze duration [i.e., the summed duration of fixations during the first-pass reading; Bertram & Hyönä, 2; Hyönä & Pollatsek, 9; Pollatsek & Hyönä, 10] with one exception [Juhasz, 11]. With shorter compound words (≤ 9 letters), the findings have been more variable. Four studies [Inhoff et al., 12; Juhasz, 13, 14; Schmidtke, Van Dyke et al., 15 see their Table S5.3] observed an effect of first-constituent frequency in gaze duration (an effect size of 33–40 ms), three [Andrews et al., 16; Bertram & Hyönä, 2; Juhasz, 11] observed a marginal effect (an effect size of 11–31 ms), and two studies [Inhoff et al., 12; Juhasz et al., 17] observed no reliable effect. With respect to Inhoff et al. [12], it should be noted that they obtained an effect of first constituent frequency for “headed compounds” (the compound head appears as the first constituent) but not for modifier-head compounds (Inhoff et al. called them “tailed compounds”).

Concerning the effects of second-constituent frequency, only one study [Pollatsek et al., 4] exists that used long compound words (an average above 12 letters) as stimuli. It found a reliable effect (95 ms) of second constituent frequency in gaze duration. The other studies used as stimuli compound words whose length was on average nine letters or less. For these short compound words, an effect in gaze duration (21–46 ms) was obtained in five studies [Inhoff et al., 12; Juhasz et al., 17; Juhasz, 13, 14; Schmidtke, Van Dyke et al., 15, see their Table S5.3], a marginal effect (15 ms in gaze duration) in one study [Andrews et al., 16]. It should be noted Inhoff et al. [12] obtained an effect of second constituent frequency for modifier-head compounds (i.e., “tailed compounds”), but not for compounds where the head was the initial constituent (i.e., “headed compounds”).

Regarding the effects of whole word frequency, all existing studies have established a reliable effect in gaze duration. Three studies [Bertram & Hyönä, 2; Juhasz, 11; Pollatsek et al., 4] used as stimuli two-constituent compounds that were on average 10.6 letters or longer. A sizeable effect of whole word frequency (79–167 ms in gaze duration) was observed for these long compounds. The effect of whole word frequency was also reliable [an effect size of 52–69 ms in gaze duration; Bertram & Hyönä, 2; Juhasz, 11, 14; Schmidtke, Van Dyke et al., 15, see their Table S5.3] for shorter compounds (an average of 6.6–8.7 letters).

The evidence reviewed above suggests that both the decomposition route and the holistic route are in operation in processing lexicalized compound words during reading. Thus, the results are consistent with the dual-route race model described above. They also provide some support for the visual acuity principle [Bertram & Hyönä, 2], according to which the decomposition route is dominant in the initial stages of processing long compounds, while the holistic route dominates the processing of short compound words (the constituent frequency effects were smaller for short than long compounds, or sometimes non-existent).

### Processing novel compound words in reading

Only few studies exist that have examined the processing of novel compound words during reading [for a review of the seminal eye-tracking studies on the effects of semantic transparency, see Hyönä, 8; for a recent eye-tracking corpus study, see Schmidtke, Van Dyke, & Kuperman, 18]. The study of Pollatsek et al. [19] is most directly related to the present study. They manipulated the frequency of the first constituent of lexicalized and novel noun-noun Finnish compounds that were on average 13 letters long. As predicted by the dual-route race model, the first constituent frequency effect was greater for novel than lexicalized compound words (the effect size in gaze duration was 92 ms for novel compounds and that for lexicalized compounds 62 ms). An analogous finding was observed in selective regression-path duration, which sums up the durations of all the fixations landing on the target word before it is exited to the right. The first constituent frequency effect was 101 ms for novel compounds and 54 ms for lexicalized compounds. On the other hand, effects of novelty did not show up in the early stages of compound word processing, as indexed by the duration of first fixation made on the word. First fixation duration only demonstrated a main effect of first constituent frequency.

Pollatsek et al. [19] explain the greater effect of constituent frequency observed for novel than lexicalized compounds to reflect meaning computation. The two nouns constituting the novel compound word has to put together to form the compound meaning. This is not always straightforward, as different thematic relations may be established between the components [e.g., Schmidtke, Gagné et al., 20]. Shoben [21] has identified 14 different thematic relations between components of English compound words. For example, the word *snow* establishes an OF relation in *snowball* (ball made of snow), but a FOR relation in *snow tire* (tire made for driving on the snow). As prototypical relations are less firmly established for infrequent than frequent first constituents, the meaning computation stage takes time for novel words, whereas this stage is typically unnecessary in reading lexicalized compounds whose meanings become readily activated as soon as its lexical representation is accessed in the mental lexicon.

A few studies have investigated how context influences the interpretation of novel noun-noun combinations during reading. Studies using a self-paced reading method have recorded reading times for larger regions (text lines or multiple-word segments) than the novel compound word itself [e.g., Gerrig & Bortfeld, 22; Middleton et al., 23]. Two eye-tracking studies [Brusnighan & Folk, 24; Cohen & Staub, 25] have employed local processing measures that more sensitively tap into the processing time course.

Brusnighan and Folk [24] presented lexicalized and novel compound words either in an informative or neutral sentence context. Here we only discuss their results for novel compound words. In addition to sentence context, they also manipulated the semantic transparency of novel compound words (e.g., *deskdoor* is an example of a semantically opaque novel compound and *drinkblend* is an example of a semantically transparent compound). The average length of the target words varied from 8.2 to 9.3 letters. When informative, the prior sentence context was supportive of the intended meaning of the novel transparent (*The party host used a blender to mix each guest a drinkblend last night*) but not of the novel opaque (*The party host used a blender to mix each guest a deskdoor last night*) compound words^1^.

In Experiment 1, Brusnighan and Folk [24] tracked readers’ eye movements when they read sentences for comprehension. In both neutral and informative context, gaze duration revealed a main effect of novelty (an effect size of 129 ms and 136 ms, respectively). In the second-pass fixation time (the summed duration of fixations returning to the target word from subsequent text regions) there was a main effect of novelty and transparency and their interaction for the informative context sentences. The interaction was due to novel opaque compounds (*deskdoor*) not fitting the context producing longer second-pass fixation times (an effect size of 202 ms) than novel transparent (*drinkblend*) compounds that fit in the context. The effect primarily reflected the greater number of regressions made to opaque than transparent novel compounds. The nature of the interaction was due to the contextual and morphological cues conflicting as to the intended interpretation of the novel compound word. In contrast, novel transparent compounds were reread for less time in informative than neutral context. The observed facilitation in processing novel transparent compounds was due to contextual and morphological cues converging on a consistent word meaning.

In sum, Experiment 1 of Brusnighan and Folk [24] demonstrated a robust novelty effect in first-pass reading: gaze durations were over 100 ms longer on novel than lexicalized compound words, regardless of whether the sentence context was neutral or informative. On the other hand, contextual cues influenced second-pass reading. Facilitation was observed in second-pass reading when the context supported the novel meaning, whereas inhibition occurred when the contextual cues were in conflict with the intended interpretation of the novel opaque compound.

The study of Cohen and Staub [25] was built on two earlier reading time studies [Gerrig & Bortfeld, 22; Middleton et al., 23]. In Experiment 1, they inserted in a neutral or a supportive context novel noun-noun compounds that are difficult to interpret out of context (e.g., *dictionary treatment*). Their reading was compared to that of easy-to-interpret adjective-noun combinations (e.g., *rough treatment*). Gaze durations on the head noun (*treatment*) were reliably longer (46 ms) when it was a part of a novel noun-noun compound word than a part of an adjective-noun phrase. More importantly, the context did not exert an effect on the first-pass reading, but only on measures indexing delayed processing. In Experiment 2, reading of the difficult-to-interpret novel compounds were compared to that of easy-to-interpret novel compounds (e.g., *dictionary treatment* vs. *torture treatment).* Gaze durations were longer (an effect size of 51 ms) on the difficult-to-interpret than the easy-to-interpret novel compounds. Again, context had no effect on the first-pass reading, but only influenced later processing. Finally, in Experiment 3 Cohen and Staub [25] strengthened the context manipulation by giving an explanation in the preceding context for the novel compound. The explanatory context also included the head noun itself. Again, the difficult-to-interpret novel compounds were read with longer gaze durations (an effect size of 28 ms) than the easy-to-interpret compounds, but again context only influenced later processing stages. Cohen and Staub [25] conclude that “some kind of default generation process operates even when a noun-noun compound is encountered in a well-developed context” (p. 163). Moreover, they suggest that their pattern of results is compatible with multistage models of conceptual combination, such as the RICE model of Spalding et al. [26], according to which meaning computation follows three stages: sense suggestion, evaluation, and elaboration. The delayed effects of context may reflect the elaboration or the evaluation stage.

Finally, we review the study of Pollatsek et al. [27] on processing novel (e.g., *unblamed*, *overmelt*) and lexicalized (e.g., *unmarried*, *overload*) prefixed derived words. The use of derivational prefixes in English (e.g., *un*- or *over*-) is productive in that novel prefixed words can be understood without supporting context. The target words were embedded in single sentences to be read for comprehension. In Experiment 1, Pollatsek et al. [27] observed a robust novelty effect (an effect size of 104 ms in gaze duration). In Experiment 2, they manipulated the frequency of word root (e.g., *melt* in *overmelt*) separately for novel and lexicalized prefixed words. Again, a robust novelty effect was obtained (an effect size of 76 ms in gaze duration). More importantly, the root frequency effect was similar in size for novel and lexicalized prefixed words (an effect size of 45 ms vs. 64 ms in gaze duration for the novel and lexicalized words, respectively).

The results of Pollatsek et al. [27] stand in contrast to those of Pollatsek et al. [19] with novel and lexicalized compound words, as the latter study observed a bigger constituent frequency effect for novel than lexicalized compound words. The discrepancy is reconciled by Pollatsek et al. [19] by assuming two levels of processing: identification of the component morphemes and meaning computation. Early constituent and root frequency effects are assumed to reflect the identification stage, which presumably works similarly for the two types of words. However, for novel prefixed words the meaning computation stage is assumed to be rule-governed (e.g., *unblamed* refers to the negation of *blamed*), so no extra time is required to relate the two morphemes with each other. On the other hand, in novel compounds multiple possible relations may be established between the constituents. As argued above, prototypical relations are less firmly established for infrequent than frequent first constituents, so the meaning computation stage takes extra time for novel words containing infrequent constituents.

### Present study

In the present study, we asked a group of competent adult readers to read for comprehension sentences that included novel and lexicalized noun-noun compound words as the targets. We orthogonally manipulated the frequency of the compound word head (i.e., the second constituent) as an independent word together with compound word length. We did this separately for novel and lexicalized compound words. We paired novel and lexicalized compound words that were similar in second constituent frequency and length and wrote a sentence frame that was identical up to the target word (for examples, see the Materials). The sentence frame preceding the target word was semantically neutral with respect to the target word meaning. On the basis of the dual-route race model [Pollatsek et al., 4] and the visual acuity principle [Bertram & Hyönä, 2], we predicted the effect of second constituent frequency to be greater for novel than lexicalized compounds, the relative difference being greater for short than long compound words. This is because short lexicalized compound words are assumed to be processed holistically; thus, demonstrating no or a negligible constituent frequency effect. On the other hand, both long lexicalized and novel compound words are assumed to be processed via the decomposition route; hence, they should both produce a robust second constituent effect.

## Methods

### Participants

Twenty-six university students participated in the study for course credit. They had normal or corrected-to-normal vision and no diagnosed reading problems. They were all native speakers of Finnish.

### Apparatus

Participants’ eye movements were recorded with EyeLink II (SR Research, Mississauga, Ontario, Canada), which is a head-mounted eye-tracker with a sampling rate of 500 Hz. There is one camera for each eye, but only the right eye was recorded. The camera was positioned 4–6 cm away from the eye so that it did not block the participant’s vision of the computer monitor where the text was displayed. The participants were seated 80 cm away from the monitor.

### Materials

Sixty existing two-constituent compound words and 60 novel two-constituent Finnish compound words were used as the target words. Word length and second constituent frequency were manipulated; on the other hand, the frequency of the first-constituent was matched across the four conditions. The short compound words were 7–9-letters long, while the long compound words were 12–16-letters long. Thirty existing and novel compounds were short and another 30 existing and novel compounds were long so that half of them (i.e., 15 words) had a low-frequency second constituent (range: 1–16 per million) and another half a high-frequency second constituent (range: 51–1143 per million). Word length was matched separately for short and long compounds. The whole word frequencies of the lexicalized compound words were low (around 1 per million; see Table 1). A 2 (word length) × 2 (second constituent frequency) analysis of variance was computed on the whole word frequency of the lexicalized compounds. No main effect of word length (*F* < 1), or the main effect of second constituent frequency (*F*(1,56) = 2.13, *p* > .1) was found. All the frequencies were derived with WordMill [Laine & Virtanen, 28] from a newspaper corpus comprising 22.7 million word tokens. All target words were noun-noun compounds. The lexical characteristics of the target words are presented in Table 1.

The novel compounds were words that did not appear in the dictionary or in the newspaper corpus. They were created by combining two existing lexemes (nouns). As word compounding is very productive in Finnish, speakers of Finnish encounter new compound words daily. Novel words were created whose meaning can be derived by combining the meaning of the component lexemes without a need for a supporting context. Their comprehensibility was tested by presenting to a group of 14 university students a set of novel compounds. They were asked to rate the comprehensibility using a 4-point scale: 1 = I know what the word means; 2 = I think I know what the word means; 3 = I have an idea what the word means; 4 = I don’t know what the word means. For the experiment, we chose novel words that had a rating of 1–3. Their average comprehensibility rating was 1.69, and the ratings ranged from 1.14 to 2.64. The ratings were comparable between the different novel compound conditions (see Table 1).

The target compounds were embedded in single sentences. They were preceded by a short neutral context that did not constrain the identity of the target word. The target words always appeared in the middle of the sentence and the text line. For each existing compound word, a matched novel compound word was selected so that they could appear in the same sentence pair. This was done separately for short and long compound words. The word pairs were matched for length and second-constituent frequency. Below, a set of example sentences and their English translations are presented for the different conditions. The target compound is presented here in bold for illustrative purposes but not in the actual experiment.

Short compound + high-frequency second constituent

Novel: *Viimeisten tietojen mukaan **savukala** ei ollut ruokamyrkytyksen aiheuttaja.*

(According to the last reports **smoked fish** was not the cause of the food poisoning.)

Lexicalized: *Viimeisten tietojen mukaan **kohusana** esiintyi aineistossa useita kertoja.*

(According to the last reports the **stir word** occurred in the material several times.)

Short compound + low-frequency second constituent

Novel: *Lomailijoita ilahdutti **eväsnakki**, joka maistui myös perheen pienimmille.*

(The vacationers were delighted by **picnic wiener** that was also liked by the children of the family.)

Lexicalized: *Lomailijoita ilahdutti **pukukoppi**, jonka sai lukkoon tavaroiden säilytystä varten.*

(The vacationers were delighted by the **changing room** that could be locked for keeping things safe.)

Long compound + high-frequency second constituent

Novel: *Työntekijät vaativat, että **tulkkitehtävä** annetaan oikealle henkilölle.*

(The employees demanded that the **translation job** will be given to the right person.)

Lexicalized: *Työntekijät vaativat, että **lounaspaikka** pitää hinnat ennallaan.*

(The employees demanded that the **lunch place** keeps the prices unchanged.)

Long compound + low-frequency second constituent

Novel: *Parhaaksi tuotteeksi valittiin **maustesivellin**, jota käytetään ruoanlaitossa.*

(As the best product people chose a **seasoning brush** that is used in cooking.)

Lexicalized: *Parhaaksi tuotteeksi valittiin **hedelmäsäilyke**, jossa on helposti irrotettava kansi.*

(As the best product people chose a **canned fruit** that has an easily removed lid.)

### Procedure

A short practice session preceded the actual experiment. In the beginning of the experiment, the eye-tracker was calibrated using a 9-point calibration grid. The 120 experimental sentences were mixed with 38 filler sentences, so each participant read altogether 158 sentences. They were asked to read the sentences silently for comprehension at their own pace. After 24 sentences, a true-false statement was presented, and the participants were asked to respond (*Yes* or *No*) whether the statement was in agreement with the last read sentence. At the beginning of each trial, a fixation cross was presented at a center-left location and the participant was asked to look at it. If needed, the calibration was adjusted, and a sentence was presented to the right of the fixation point. After completing reading the sentence, the participant pressed a key in the keyboard to proceed to the next trial or to the sentence verification. The sentences appeared in Courier New font (font size 18) as black against a white background.

The experimental sentences were divided into two blocks so that the two members of each sentence pair appeared in different blocks. In each block, there was an equal number of sentences across the 8 experimental conditions. Within the block, the order of sentences was randomized. The order of blocks was counterbalanced across participants. There was a short pause between the blocks.

The eye-tracker was recalibrated prior to the presentation of the second block. The experiment lasted for about 50 minutes.

## Results

### Data preparation

Word-level eye movement measures for the target word were computed from the eye movement data. *First-fixation duration* is the duration (in ms) of the initial fixation that a reader makes in a word. *Gaze duration* is the duration of all fixations in the target word until the eyes fixate away from it either to the left of right. *Selective regression-path duration* is the sum of fixations and re-fixations of the target word before it is exited to the right. *Total fixation time* is the sum of all the fixations made on the target word. *Probability of look-backs* is the likelihood the target word is looked back to after exiting it.

The reading time measures were skewed and consequently transformed. For all the measures, square root transformation was the best fitting transformation to normalize them. Observed means and standard deviations of the eye movement measures are presented in Table 2. The data and materials are available at https://osf.io/5v9xm/.

### Statistical analyses

Data were analyzed with linear mixed-effects models (LMM) using the *lme4* package [Bates et al., 29] in the R statistical software [Version 3.6.2; R Core Team, 30]. Separate models were built for each eye movement measure. It has been recommended that only minimal data filtering is performed when analyzing data with mixed-effects models, combined with model criticism [Baayen & Milin, 31]. We compared models using non-filtered fixation times to filtered models, in which reading times > |2.5| *SD* were filtered out. It turned out that R^2^ values were virtually identical between the filtered and non-filtered models, and none of the observed effects changed. Thus, models based on non-filtered values are reported below.

Word type (novel vs. real), frequency of 2^nd^ constituent (high vs. low) and word length (short vs. long) were fitted to each model as deviation coded fixed effects variables. Participants and items were entered to the models as random intercepts [Baayen et al., 32]. Word type, 2^nd^ constituent frequency, word length and their interactions were fitted as by-subject random slopes [see Brauer & Curtin, 33]. If the model failed to converge after fitting the full random structure, the random structure of the model was trimmed top-down, starting with removing correlations between factors.

The exact degrees of freedom are difficult to determine for the *t*-statistics estimated by LMMs, leading to problems in determining exact *p*-values [Baayen et al., 32]. Consequently, degrees of freedom or p-values are not reported; statistical significance at the .05 level is indicated by values of the | *t* or *z* | > 1.96. For the sake of brevity, only significant effects are reported in the text. The final models are reported in Tables A1–A5 in the Appendix. Below, we report the eye movement measures in the order that reflects the time line of processing, starting with first fixation duration, followed by gaze duration, selective regression-path duration, total fixation time, and probability of look-backs.

#### First fixation duration

**Figure 1.**
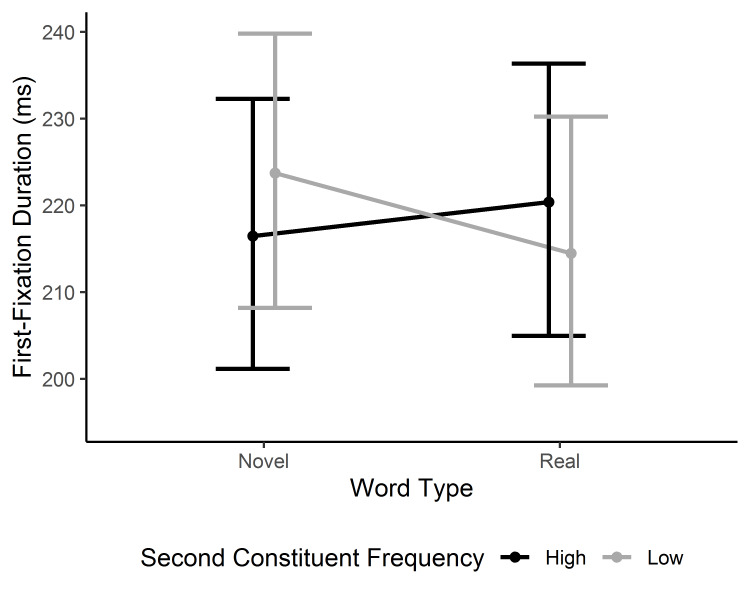
Model estimates for the first-fixation duration in the target word. The model means and confidence intervals are back-transformed from square-root transformed values. Error bars represent 95% CI.

The analysis of first fixation duration showed no main effect of novelty. However, an interaction between word type and 2^nd^ constituent frequency was observed, β = -0.44, 95% CI [-0.81, -0.08], *t* = 2.38 (see Figure 1). The results indicate a novelty effect for low-frequency 2^nd^ constituent words: first-fixation duration was shorter for existing than novel compound words. On the other hand, existing and novel compound words with high-frequency 2^nd^ constituents were read similarly. Word length did not modify this interaction; however, a reliable main effect of length was established, β = -0.55, 95% CI [-0.81, -0.29], *t* = -4.09. First fixation duration for short words was longer than that for long words. It likely reflects the fact that long words more likely received a refixation on the word. A single fixation tends to be longer than the first of multiple fixations.

#### Gaze duration

**Figure 2.**
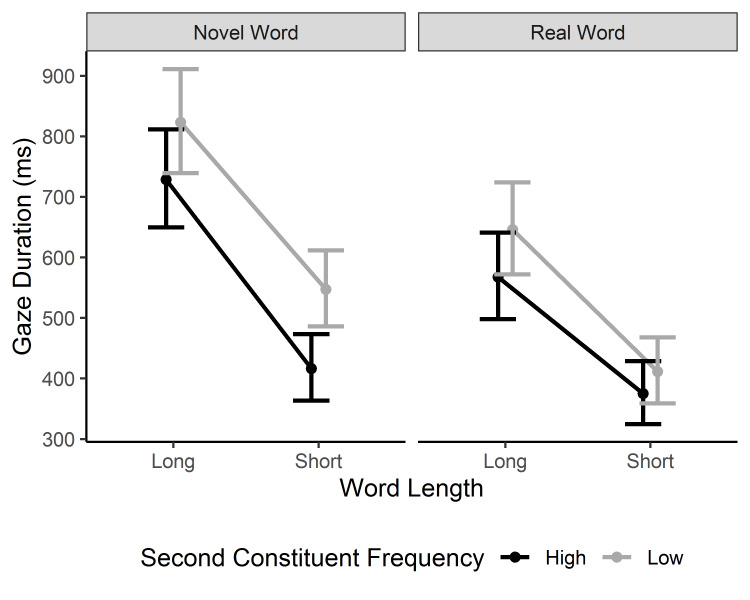
Model estimates for the gaze duration on the target word. The model means and confidence intervals are back-transformed from square-root transformed values. Error bars represent 95% CI.

The model on gaze duration revealed all three main effects. Moreover, the two-way interactions of Word Type × Word Length (a bigger length effect for novel words) and Word Type × 2^nd^ Constituent Frequency (a bigger constituent frequency effect for novel compounds) were modified by a three-way interaction between word type, 2^nd^ constituent frequency, and length, β = 1.96, 95% CI [0.10, 2.90], *t* = 2.40 (see Figure 2). The three-way interaction indicates that the constituent frequency effect was of similar size for long novel and lexicalized compound words, whereas it was much bigger for short novel than lexicalized compounds. For short lexicalized compounds, the constituent frequency effect was virtually non-existent, as evidenced by the confidence intervals of the estimated means of the two frequency conditions significantly overlapping (see Figure 2). However, it should be noted that in the observed means (see Table 2) there is a 42-ms second constituent frequency effect in gaze/ ableuration for the short lexicalized compounds.

#### Selective regression-path duration

**Figure 3.**
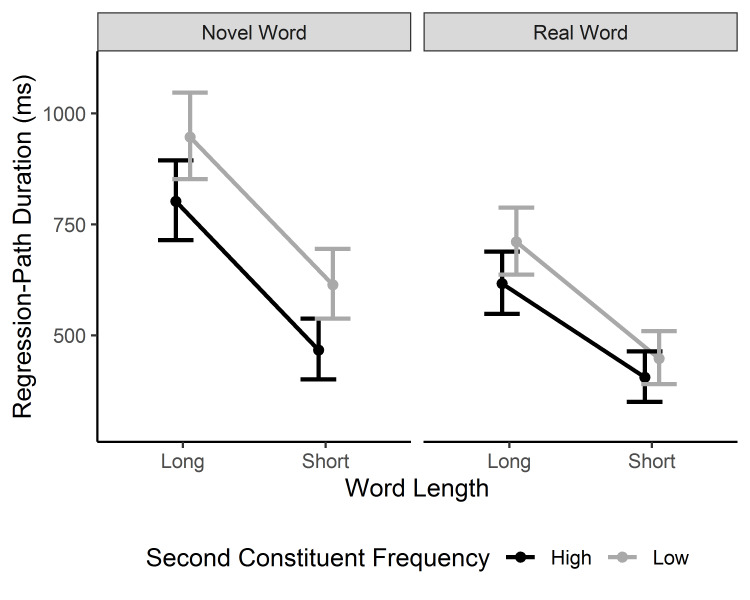
Model estimates for the selective regression-path duration in the target word. The model means and confidence intervals are back-transformed from square-root transformed values. Error bars represent 95% CI.

The overall pattern of results for selective regression-path duration closely mimicked that for gaze duration. The three main effects were modified by the two-way interactions of Word Type × Word Length (a bigger length effect for novel words) and Word Type × 2^nd^ Constituent Frequency (a bigger constituent frequency effect for novel compounds), which were further modified by a three-way interaction between word type, 2nd constituent frequency, and length, β = 1.50, 95% CI [0.10, 2.90], *t* = 2.09 (see Figure 3). The three-way interaction indicates that the constituent frequency effect was bigger for novel than lexicalized compounds, particularly when they were short. For novel words, the second constituent frequency effect was greater for short than long words. Moreover, short novel compounds displayed a sizeable constituent frequency effect, whereas short existing compounds demonstrated virtually no effect of 2^nd^ constituent frequency.

#### Total fixation time

**Figure 4.**
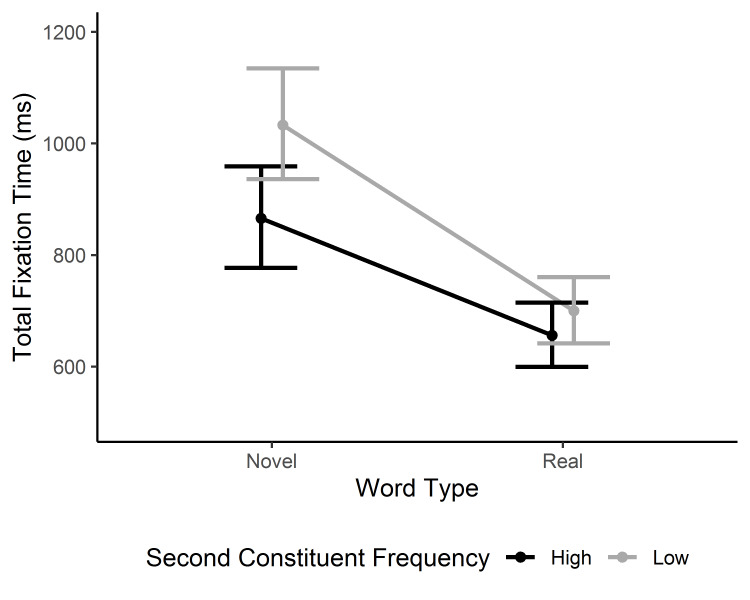
Model estimates for the total fixation time in the target word. The model means and confidence intervals are back-transformed from square-root transformed values. Error bars represent 95% CI.

Some of the effects apparent in gaze duration and selective regression-path duration were washed away in total fixation time reflecting more delayed processing. Apart from the three main effects, there was only an interaction between word type and 2^nd^ constituent frequency, β = -1.87, 95% CI [-2.73, -1.02], *t* = -4.28 (see Figure 4). The interaction reflects a greater constituent frequency effect for novel than existing words.

#### Probability of look-backs

**Figure 5.**
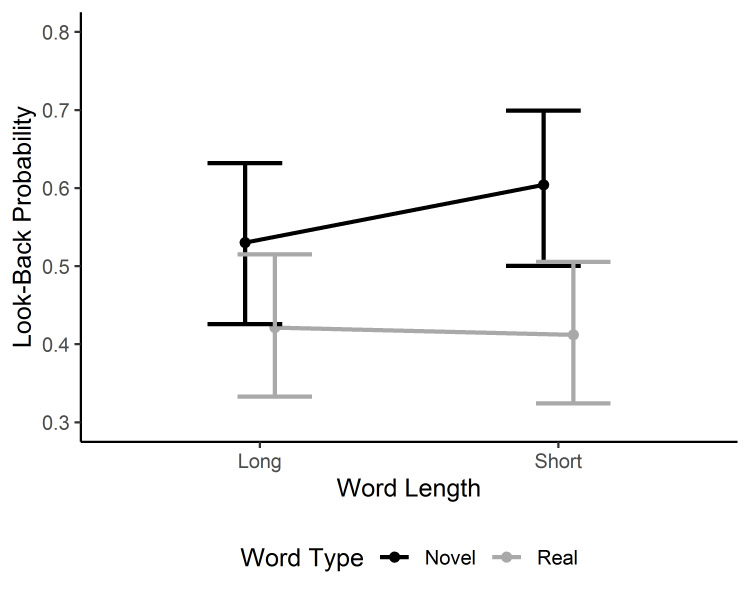
Model estimates for the probability of look-backs to the target word. Error bars represent 95% CI.

The most delayed effects are reflected in the probability of looking back to the target word after exiting it. The model on the probability of look-backs revealed a main effect of word type, indicating that readers were more likely to return to novel than lexicalized compound words. This main effect was qualified by an interaction between word type and word length, β = -0.34, 95% CI [-0.65, -0.03], z = -2.18. The result indicates that the probability of look-backs was greater for novel short than long words, whereas lexicalized compound words showed virtually no effect of word length (see Figure 5).

## Discussion

In the present study, we investigated the processing of novel and lexicalized compound words when they appeared in a neutral sentence context. Readers’ eye fixations on the target words were recorded while they read the sentences silently for comprehension. Based on the dual-route race model [Pollatsek et al., 4] and the visual acuity principle [Bertram & Hyönä, 2; Hyönä, 34], we predicted novel compounds and long lexicalized compounds to be recognized via the (de)composition route, while the short lexicalized compounds would be recognized via the holistic route as single entities. The degree of compositional processing was assessed by manipulating the frequency of the compound word head (i.e., the second constituent) separately for long and short novel and lexicalized compounds. According to the logic [Taft & Forster, 6], the greater the component frequency effect, the stronger the involvement of the decomposition route.

The results were consistent with the predictions. First, the time spent on the target word during first-pass reading (gaze duration) was generally longer for novel than lexicalized compound words. Second and more importantly, the second constituent frequency effect in gaze duration and selective regression-path duration was greater for novel than lexicalized compound words. It suggests that the (de)composition route was more strongly in operation when reading novel than lexicalized compound words. Third, in gaze duration and selective regression-path duration the difference between novel and lexicalized compounds in the size of the second constituent frequency effect was particularly noticeable for short compounds. For short lexicalized compounds, the effect was negligible, but a robust second constituent frequency effect was observed for short novel compounds. A robust second constituent frequency effect was also observed for long compounds (both novel and lexicalized). This pattern of results is generally consistent with the visual acuity principle, which posits that short lexicalized compound words are processed via the holistic route, whereas with long compounds recognition is initiated using the decomposition route. Finally, in the measures capturing most delayed effects, the three-way interaction was no longer reliable. The delayed measures indicated that readers looked back to novel compounds more than to lexicalized compounds, especially when the novel words were short. Moreover, the total fixation time was longer on novel than lexicalized compounds, especially when the second constituent was infrequent. The nature of the observed delayed effects was not exactly as predicted.

Eye movement studies similar to the present study have been criticized for inflating the risk of Type 1 error by analyzing multiple dependent measures to study how the processing evolves over time [von der Malsburg & Angele, 35]. One possible solution for this is the use of the rule-of-thumb criterion, i.e., if the same effect is found in more than one fixation time measure, it is likely to be reliable [von der Malsburg & Angele, 35]. The two-way interaction between word type and second constituent frequency was observed in three measures (gaze duration, selective regression-path duration, total fixation time) and the three-way interaction between word length, word type and second constituent frequency was observed in two measures (gaze duration and selective regression-path duration). Thus, using the rule-of-thumb criterion, these most important results of the present study can be considered reliable. The same is true for all the main effects.

For long compounds, the pattern of results is consistent with that obtained by Pollatsek et al. [19] who manipulated the frequency of the first constituent for long novel and lexicalized compounds. Similar to their study, the model estimates for gaze duration and selective regression-path duration suggest a greater second constituent frequency effect for long novel than lexicalized compounds. This tendency is not as clear in the observed means. The novelty effects observed in the present study are also consistent with those reported by Brusnighan and Folk [24] for reading novel and lexicalized English compounds in neutral context. Brusnighan and Folk [24] reported a robust novelty effect in gaze duration. They are also consistent with the results of Cohen and Staub [25]. In Experiment 1, they compared reading of novel spaced compounds (dictionary treatment) to that of adjective-noun phrases (rough treatment). Gaze duration on the head noun was found to be longer for novel compounds than adjective-noun phrases. However, it should be noted that Cohen and Staub [25] studied novel compounds that were difficult to interpret out of context, whereas in the present study the novel compounds were interpretable without supporting context.

### Theoretical considerations regarding the recognition of novel compound words

Above, following the logic of Taft and Forster [6], we have interpreted the bigger second constituent frequency effect observed for novel than lexicalized compounds to suggest a stronger involvement of the decomposition route in reading novel compound words. However, as argued by Pollatsek et al. [19], to understand novel compounds, it is not sufficient to recognize the constituent lexemes as separate words via the decomposition process, but the constituent meanings need to be put in relation with each other to compute the novel meaning. Thus, the observed processing differences between novel and lexicalized compounds are likely to not only reflect a greater involvement of the decomposition route, but also a need for meaning computation that is unnecessary in processing lexicalized compounds.

The stronger second constituent frequency effect in reading novel than lexicalized compounds is readily interpretable as a reflection of computational processing. When the head noun is infrequent in the language as a separate word, meaning computation is more demanding than it is the case when the head noun is frequent in the language. This interpretation is consistent with the *morphological transcendence hypothesis* put forth by Libben [36]. His main claim is that compound word constituents are position-bound representations with distinct probabilities and identities as modifiers or heads. By exposure to written and spoken language, constituent meanings emerging in language users’ minds become non-identical to their meaning as separate words. Thus, the lexeme *key* has a somewhat different meaning as a modifier (e.g., *keyboard*), a head (e.g., *room key*) and a separate word. In other words, constituent meanings as modifiers and heads are constrained and influenced by their respective constituent families [see also Kuperman et al., 37; Sun et al., 38]. Thus, as infrequent words are less likely to form compound constituents, processing novel compounds that contain infrequent words as heads (e.g., *-leike* = cutlet in *savuleike* = smoked cutlet) is expected to be more time-consuming than that with frequent words (e.g., *-tuote* = product in *sikatuote* = pork product) as heads. As noted above, such result was obtained in the present study. However, with respect to lexicalized compounds, the morphological transcendence hypothesis cannot readily explain the observed modulation of the constituent frequency effect by word length. It does not ascribe any significance to compound word length. Yet, as noted above, the word length modulation is readily interpretable by the visual acuity principle [Bertram & Hyönä, 2].

In future studies, the morphological transcendence hypothesis of Libben [36] can be tested by simultaneously manipulating the constituent frequency as a modifier or a head versus a separate word. The hypothesis would be supported by data demonstrating that constituent frequencies as modifiers / heads predict better compound word processing than constituent frequencies as separate words.

The robust constituent frequency effect obtained for novel compounds may also be understood within the *RICE framework* of Spalding et al. [26]. This theory has been proposed to explain meaning generation of novel compound words. The key assumption is that meaning generation is not a straightforward process, as component meanings can be brought in relation with each other in multiple ways [e.g., Gagné & Spalding, 39]. For example, to comprehend what *maustesivellin* (seasoning brush) means the reader has to relate the component meanings to each other to grasp that it refers to a brush used for spreading seasoning in cooking, rather than, for example, a brush made of seasoning. According to RICE, meaning generation is initiated by the modifier suggesting possible relations, with which it can be linked to the head. Head-based relational information and context is then used to evaluate the activated relations and choose the correct interpretation. If necessary, the generated meaning can subsequently be further elaborated. In their recent computational model [*CAOSS*; Marelli et al., 40], the symbolic relations between constituents postulated in RICE are replaced in CAOSS by data-driven weight patterns.

Even though the present results are broadly in line with the *RICE framework*, the present study cannot be considered a direct test of the theory. This is because the present study did not tap into the process of how exactly the components are related to each other to generate meanings for novel words. For example, no contextual manipulation was applied to constrain possible relations between the component meanings. Thus, we cannot make specific claims about the exact nature of meaning generation. What we can say is that meaning generation has an impact already on the first-pass reading of novel compounds. Yet, the effect lingers on, as evidenced by the regression-path duration measure. As the prior context was completely neutral with respect to the word meaning, regressive fixations were most likely executed to give the reader additional time to compute a meaning for the novel compounds, rather than searching for additional information to constrain the generated meaning.

### Conclusions

The present study provides further evidence for the parallel dual-route model of compound word processing [Pollatsek et al., 4] and the visual acuity principle [Bertram & Hyönä, 2]. In line with predictions derived from these notions, the decomposition route is more strongly utilized in processing novel than lexicalized compound words, particularly when they are short. On the other hand, the holistic route prevails in processing short lexicalized compounds words. This is because short compound words fit in the foveal area of the eyes where the visual acuity is at its best; hence, they can be processed as single entities. Finally, it is argued that the bigger second constituent frequency effect obtained for novel compounds not only reflect morphological decomposition, but also meaning computation.

## Figures and Tables

**Table 1.** Lexical characteristics of the target words

Characteristic	Long compound words				Short compound words			
	LF 2^nd^ const.		HF 2^nd^ const.		LF 2^nd^ const.		HF 2^nd^ const.	
	Existing	Novel	Existing	Novel	Existing	Novel	Existing	Novel
Word length^a^	14.27	14.27	13.4	13.87	8.47	8.2	8.13	8.13
2^nd^ const. length^a^	7.33	7.27	6.13	6.93	4.4	4.53	4.13	4
1^st^ const. frequency^b^	24	27	19	19	34	27	27	29
2^nd^ const. frequency^b^	3.7	3.8	471	432	4.1	4.8	403	450
Word frequency^b^	0.53	0	1.23	0	0.75	0	1.45	0
Comprehensibility^c^		1.53		1.63		1.92		1.71

Note. LF = low frequency; HF = high frequency; ^a^ = in letters; ^b^ = per million words; ^c^ = 4-point scale, where 1 = I know what the word means and 4 = I don’t know what the word means

**Table 2.** Descriptive statistics for the eye fixation measures

			Word Type			
			Novel		Real	
Measure	Word Length	2nd Constituent Frequency	*M*	*SD*	*M*	*SD*
First-fixation duration	Long	High	233	82	237	77
		Low	247	79	235	74
	Short	High	219	105	220	96
		Low	221	128	210	96
Gaze Duration	Long	High	772	367	607	322
		Low	883	456	698	397
	Short	High	455	284	396	182
		Low	610	414	438	217
Selective regression-path duration	Long	High	836	352	550	310
		Low	991	438	752	379
	Short	High	507	298	426	187
		Low	651	407	473	219
Total fixation time	Long	High	1090	519	836	411
		Low	1238	538	903	542
	Short	High	763	424	567	304
		Low	958	548	604	321
Probability of look-backs	Long	High	0.53	0.50	0.48	0.50
		Low	0.51	0.50	0.39	0.49
	Short	High	0.58	0.49	0.43	0.50
		Low	0.58	0.49	0.42	0.49

## Footnotes

^1^ It is unfortunate that they did not have a condition where the context would have been informative of the novel opaque compounds and uninformative of novel transparent compounds.

## Appendix

### Final models for each eye fixation measure

**Table A1.** Final model for first fixation duration.

Random effects	*n*	*Variance*	*SD*
Participants (Intercept)	27	0.08	0.29
Word (Intercept)	60	1.65	1.29
Participants (Length)		0.10	0.31
Residual		7.10	2.66
Fixed effects	β	95 % CI	*t*
Intercept	14.79	14.29 – 15.29	**58.10**
Word Type (Existing vs. Novel)	0.09	-0.09 – 0.27	0.96
Second Constituent Frequency	-0.02	-0.26 – 0.21	-0.19
Length	-0.55	-0.81 – -0.29	**-4.09**
Word Type × Second Constituent Frequency	-0.44	-0.81 – -0.08	**-2.38**
Word Type × Length	0.14	-0.22 – 0.51	0.76
Frequency × Length	0.25	-0.22 – 0.71	1.02
Word Type × Second Constituent Frequency × Length	0.12	-0.61 – 0.86	0.33

Note. The *t-*values > 1.96 are in boldface. Number of observations = 3240.

**Table A2.** Final model for gaze duration.

Random effects	*n*	*Variance*	*SD*
Participants (Intercept)	27	2.42	1.56
Word (Intercept)	60	7.43	2.73
Participants (Length)		0.80	0.89
Residual		33.56	5.79
Fixed effects	β	95 % CI	*t*
Intercept	23.54	22.42 – 24.66	**41.24**
Word Type (Existing vs. Novel)	2.65	2.25 – 3.05	**13.01**
Second Constituent Frequency	-1.8	-2.68 – -0.92	**-4.00**
Length	5.36	4.42 – 6.31	**11.13**
Word Type × Second Constituent Frequency	-1.08	-1.88 – -0.28	**-2.66**
Word Type × Length	1.14	0.35 – 1.95	**2.83**
Frequency × Length	0.31	-1.46 – 2.07	0.34
Word Type × Second Constituent Frequency × Length	1.96	0.36 – 3.55	**2.40**

Note. The *t-*values > 1.96 are in boldface. Number of observations = 3240.

**Table A3.** Final model for selective regression-path duration.

Random effects	*n*	*Variance*	*SD*
Participants (Intercept)	27	3.12	1.77
Word (Intercept)	60	8.24	2.87
Participants (Word Type)		1.04	1.02
Residual		26.02	5.10
Fixed effects	β	95 % CI	*t*
Intercept	24.78	23.60 – 25.96	**41.00**
Word Type (Existing vs. Novel)	3.17	2.65 – 3.70	**11.94**
Second Constituent Frequency	-2.12	-3.08 – 1.16	**-4.32**
Length	5.73	4.72 – 6.74	**11.70**
Word Type × Second Constituent Frequency	-1.38	-2.08 – -0.68	**-3.85**
Word Type × Length	1.25	0.55 – 1.96	**3.50**
Frequency × Length	-0.04	-1.96 – 1.88	-0.04
Word Type × Second Constituent Frequency × Length	1.50	0.10 – 2.90	**2.09**

Note. The *t-*values > 1.96 are in boldface. Number of observations = 3240.

**Table A4.** Final model for total fixation time.

Random effects	*n*	*Variance*	*SD*
Participants (Intercept)	27	3.33	1.82
Word (Intercept)	60	7.78	2.79
Participants (Word Type)		2.96	1.72
Residual		38.84	6.23
Fixed effects	β	95 % CI	*t*
Intercept	28.41	27.24 – 29.57	**47.64**
Word Type (Existing vs. Novel)	4.75	3.97 – 5.53	**11.97**
Second Constituent Frequency	-1.78	-2.80 – -0.76	**-3.43**
Length	5.05	4.03 – 6.07	**9.72**
Word Type × Second Constituent Frequency	-1.87	-2.73 – -1.02	**-4.28**
Word Type × Length	-0.03	-0.89 – 0.83	-0.07
Frequency × Length	0.47	-1.56 – 2.51	0.46
Word Type × Second Constituent Frequency × Length	1.16	-0.55 – 2.88	1.32

Note. The *t-*values > 1.96 are in boldface. Number of observations = 3240.

**Table A5.** Final model for the probability to initiate a look-back.

Random effect	*n*	*Variance*	*SD*
Participants (Intercept)	27	0.09	0.29
Word (Intercept)	60	0.67	0.82
Participants (Frequency)		0.04	0.21
Residual		1.39	1.18
Fixed effect	β	95 % CI	*z*
Intercept	-0.03	-0.41 – 0.34	-0.17
Word Type (Real vs Novel)	0.61	0.45 – 0.77	**7.40**
Frequency	0.15	-0.07 – 0.38	1.35
Length	-0.13	-0.36 – 0.09	-1.17
Word Type × Frequency	-0.19	-0.50 – 0.11	-1.24
Word Type × Length	-0.34	-0.65 – -0.03	**-2.18**
Frequency × Length	0.25	-0.19 – 0.70	1.13
Word Type × Frequency × Length	-0.24	-0.85 – 0.37	-0.77

Note. The *t-v*alues > 1.96 are in boldface. Number of observations = 3240.
